# Supplementary material for: Different Impact of Heart Rate Variability in the Deep Cerebral and Central Hemodynamics at Rest: An in silico Investigation
Source: Front Neurosci. 2021 May 17;15:600574. doi: 10.3389/fnins.2021.600574 (PMC8165247; doi:10.3389/fnins.2021.600574)
Supplement: Supplementary file 1 [file Data_Sheet_1.pdf]

frontiers

Supplementary Material
Different impact of heart rate variability in the deep cerebral and central hemodynamics at rest: an *in silico* investigation

Stefania Scarsoglio [1,*], and Luca Ridolfi [2]

[1] *Department of Mechanical and Aerospace Engineering, Politecnico di Torino, Torino, Italy*
[2] *Department of Environmental, Land and Infrastructure Engineering, Politecnico di Torino, Torino, Italy*

Correspondence*:
Stefania Scarsoglio
stefania.scarsoglio@polito.it

A full description of the cardiovascular-cerebral model is provided, together with parameter values and initial conditions adopted for the healthy and heart failure conditions.

CARDIOVASCULAR MODEL

The cardiovascular model proposed by [1–3] is coupled to a baroreceptor model [4] to describe the whole (arterial and venous) circulation system in presence of short-term autoregulation. The model is composed by a network of compliances, C, resistances, R, and inductances, L, representing the pumping heart coupled to the systemic and pulmonary systems. The viscous effects are taken into account by the resistances, R [mmHg s/ml], the inertial terms are considered by the inductances, L [mmHg s^2/ml], while the elastic vessel properties are described by the compliances, C [ml/mmHg]. Three cardiovascular variables are involved at each section: the blood flow, Q [ml/s], the volume, V [ml], the pressure, P [mmHg].

All the four chambers of the pumping heart are described as active. The pulsatility properties are included by means of two pairs of time-varying elastance functions, one for the atria and one for the ventricles, which are then used in the constitutive equations (relating pressure, P, and volume, V). For the four heart valves, the basic pressure-flow relation is described by an orifice model. The valve motion mechanisms are deeply analyzed and account for the following blood-flow effects: pressure difference across the valve, frictional effects from neighboring tissue resistance, the dynamic motion effect of the blood acting on the valve leaflet, the action of the vortex downstream of the valve. An equation for the mass conservation (accounting for the volume variation, dV/dt) concludes the description of each chamber.

The systemic and pulmonary systems are divided into five sections. The systemic arteries are described by four sections (aortic sinus, artery, arteriole and capillary), while the systemic venous circulation is characterized by a unique compartment. The pulmonary circuit follows the same architecture. Each section of the systemic and pulmonary circuits may contain three components (the viscous term, R, the inertial term, L, and the elastic term, C), and is characterized by three equations: an equation of motion (accounting for the flow variation, dQ/dt), an equation for the conservation of mass (expressed in terms of pressure variations, dP/dt), and a linear state equation between pressure and volume.

The baroreceptor model combines the afferent and the central nervous system into a unified description, so that the sympathetic, n_s, and parasympathetic, n_p, activities are directly related to the carotid sinus pressure through a steady state sigmoidal function. The efferent responses consist of a static component, which is a linear combination of n_s and n_p, and a dynamic component, which is a first order ordinary differential equation in time. The short-term baroregulation accounts for the inotropic effect of both ventricles (neglecting for simplicity the parasympathetic influence), as well as the control of the systemic vasculature (peripheral arterial resistances, unstressed volume of the venous system, and venous compliance). The chronotropic effects due to the heart rate regulation are instead implicitly and intrinsically taken into account by the RR extraction.

Equations

For the sake of simplicity, the equations are grouped into cardiac (with the four chambers), circulatory (with systemic and pulmonary loops), and baroreceptor mechanism sections.

Left atrium

$$\begin{aligned}
\frac{dV_{la}}{dt} &= Q_{pvn} - Q_{mi}, \\
P_{la} &= P_{la,un} + E_{la}(V_{la} - V_{la,un}), \\
Q_{mi} &= \begin{cases} CQ_{mi}AR_{mi}\sqrt{P_{la} - P_{lv}}, & \text{if} \;\; P_{la} \geq P_{lv}, \\ -CQ_{mi}AR_{mi}\sqrt{P_{lv} - P_{la}}, & \text{if} \;\; P_{la} < P_{lv}, \end{cases}
\end{aligned} \tag{S1}$$

where the subscript un denotes the unstressed pressure and volume levels of each cardiovascular section. The time-varying elastance is

$$E_{la}(t) = E_{la,min} + \frac{E_{la,max} - E_{la,min}}{2} e_a(t), \tag{S2}$$

and the atrium activation function is

$$e_a(t) = \begin{cases} 0, & \text{if} \;\; 0 \leq t \leq T_{ac}, \\ 1 - \cos\left(\frac{t - T_{ac}}{RR - T_{ac}} 2\pi\right), & \text{if} \;\; T_{ac} < RR, \end{cases} \tag{S3}$$

where $E_{la,min}$ and $E_{la,max}$ are the minimum and maximum elastance values, respectively, while T_{ac} is the beginning of atrial contraction.

The valve opening is decided by the angular position of the leaflets:

$$AR_{mi} = \frac{(1 - \cos\theta_{mi})^2}{(1 - \cos\theta_{max})^2} \tag{S4}$$

and the valve motion is governed by

$$\frac{d^2\theta_{mi}}{dt^2} = \begin{cases} (P_{la} - P_{lv})K_{p,mi}\cos\theta_{mi} - K_{f,mi}\frac{d\theta_{mi}}{dt} \\ +K_{b,mi}Q_{mi}\cos\theta_{mi} \\ -K_{v,mi}Q_{mi}\sin 2\theta_{mi}, \quad \text{if} \;\; Q_{mi} \geq 0, \\ (P_{la} - P_{lv})K_{p,mi}\cos\theta_{mi} - K_{f,mi}\frac{d\theta_{mi}}{dt} \\ +K_{b,mi}Q_{mi}\cos\theta_{mi}, \quad \text{if} \;\; Q_{mi} < 0, \end{cases} \tag{S5}$$

Left ventricle

$$\begin{aligned} \frac{dV_{lv}}{dt} &= Q_{mi} - Q_{ao}, \\ P_{lv} &= P_{lv,un} + E_{lv}(V_{lv} - V_{lv,un}), \\ Q_{ao} &= \begin{cases} CQ_{ao}AR_{ao}\sqrt{P_{lv} - P_a}, & \text{if} \;\; P_{lv} \geq P_a, \\ -CQ_{ao}AR_{ao}\sqrt{P_a - P_{lv}}, & \text{if} \;\; P_{lv} < P_a, \end{cases} \end{aligned} \tag{S6}$$

The time-varying elastance is

$$E_{lv}(t) = E_{lv,min} + \frac{E_{lv,max} - E_{lv,min}}{2} e_v(t), \tag{S7}$$

and the ventricle activation function is

$$e_v(t) = \begin{cases} 1 - \cos\left(\frac{t}{T_{me}}\pi\right), & \text{if} \;\; 0 \leq t < T_{me}, \\ 1 + \cos\left(\frac{t - T_{me}}{T_{ce} - T_{me}}\pi\right), & \text{if} \;\; T_{me} \leq t < T_{ce} \\ 0, \quad \text{if} \;\; T_{ce} \leq t < RR, \end{cases} \tag{S8}$$

where $E_{lv,min}$ and $E_{lv,max}$ are the minimum and maximum elastance values, respectively. T_{me} and T_{ce} are the instants where the elastance reaches its maximum and constant values, respectively.

The valve opening is decided by the angular position of the leaflets:

$$AR_{ao} = \frac{(1 - \cos\theta_{ao})^2}{(1 - \cos\theta_{max})^2} \tag{S9}$$

and the valve motion is governed by

$$\frac{d^2\theta_{ao}}{dt^2} = \begin{cases} (P_{lv}-P_a)K_{p,ao}\cos\theta_{ao} - K_{f,ao}\frac{d\theta_{ao}}{dt} \\ \quad +K_{b,ao}Q_{ao}\cos\theta_{ao} \\ \quad -K_{v,ao}Q_{ao}\sin 2\theta_{ao}, \quad \text{if} \quad Q_{ao}\geq 0, \\ (P_{lv}-P_a)K_{p,ao}\cos\theta_{ao} - K_{f,ao}\frac{d\theta_{ao}}{dt} \\ \quad +K_{b,ao}Q_{ao}\cos\theta_{ao}, \quad \text{if} \quad Q_{ao}<0, \end{cases} \tag{S10}$$

Right atrium

$$\begin{aligned} \frac{dV_{ra}}{dt} &= Q_{svn} - Q_{ti}, \\ P_{ra} &= P_{ra,un} + E_{ra}(V_{ra} - V_{ra,un}), \\ Q_{ti} &= \begin{cases} CQ_{ti}AR_{ti}\sqrt{P_{ra}-P_{rv}}, & \text{if} \quad P_{ra}\geq P_{rv}, \\ -CQ_{ti}AR_{ti}\sqrt{P_{rv}-P_{ra}}, & \text{if} \quad P_{ra}<P_{rv}, \end{cases} \end{aligned} \tag{S11}$$

The time-varying elastance is

$$E_{ra}(t) = E_{ra,min} + \frac{E_{ra,max}-E_{ra,min}}{2}e_a(t), \tag{S12}$$

where the activation function is given by Eq. (S3), while $E_{ra,min}$ and $E_{ra,max}$ are the minimum and maximum elastance values, respectively.

The valve opening is decided by the angular position of the leaflets:

$$AR_{ti} = \frac{(1-\cos\theta_{ti})^2}{(1-\cos\theta_{max})^2} \tag{S13}$$

and the valve motion is governed by

$$\frac{d^2\theta_{ti}}{dt^2} = \begin{cases} (P_{ra}-P_{rv})K_{p,ti}\cos\theta_{ti} - K_{f,ti}\frac{d\theta_{ti}}{dt} \\ \quad +K_{b,ti}Q_{ti}\cos\theta_{ti} \\ \quad -K_{v,ti}Q_{ti}\sin 2\theta_{ti}, \quad \text{if} \quad Q_{ti}\geq 0, \\ (P_{ra}-P_{rv})K_{p,ti}\cos\theta_{ti} - K_{f,ti}\frac{d\theta_{ti}}{dt} \\ \quad +K_{b,ti}Q_{ti}\cos\theta_{ti}, \quad \text{if} \quad Q_{ti}<0, \end{cases} \tag{S14}$$

Right ventricle

$$
\begin{aligned}
\frac{dV_{rv}}{dt} &= Q_{ti} - Q_{po}, \\
P_{rv} &= P_{rv,un} + E_{rv}(V_{rv} - V_{rv,un}), \\
Q_{po} &= \begin{cases} CQ_{po}AR_{po}\sqrt{P_{rv} - P_{pas}}, & \text{if} \quad P_{rv} \geq P_{pas}, \\ -CQ_{po}AR_{po}\sqrt{P_{pas} - P_{rv}}, & \text{if} \quad P_{pas} > P_{rv}, \end{cases}
\end{aligned}
\tag{S15}
$$

The time-varying elastance is

$$
E_{rv}(t) = E_{rv,min} + \frac{E_{rv,max} - E_{rv,min}}{2} e_v(t),
\tag{S16}
$$

where the ventricle activation function is given by Eq. (S8), while $E_{rv,min}$ and $E_{rv,max}$ are the minimum and maximum elastance values, respectively.

The valve opening is decided by the angular position of the leaflets:

$$
AR_{po} = \frac{(1 - \cos\theta_{po})^2}{(1 - \cos\theta_{max})^2}
\tag{S17}
$$

and the valve motion is governed by

$$
\frac{d^2\theta_{po}}{dt^2} = \begin{cases} (P_{rv} - P_{pas})K_{p,po}\cos\theta_{po} - K_{f,po}\frac{d\theta_{po}}{dt} \\ +K_{b,po}Q_{po}\cos\theta_{po} \\ -K_{v,po}Q_{po}\sin 2\theta_{po}, \quad \text{if} \quad Q_{po} \geq 0, \\ (P_{rv} - P_{pas})K_{p,po}\cos\theta_{po} - K_{f,po}\frac{d\theta_{po}}{dt} \\ +K_{b,po}Q_{po}\cos\theta_{po}, \quad \text{if} \quad Q_{po} < 0, \end{cases}
\tag{S18}
$$

Systemic Circuit

$$
\begin{cases} \frac{dP_a}{dt} = \frac{Q_{ao} - Q_{sas}}{C_{sas}}, \\ \frac{dQ_{sas}}{dt} = \frac{P_a - P_{sat} - R_{sas}Q_{sas}}{L_{sas}}, \\ P_a - P_{sas,un} = \frac{1}{C_{sas}}(V_{sas} - V_{sas,un}), \end{cases}
\tag{S19}
$$

$$\begin{cases} \frac{dP_{sat}}{dt} = \frac{Q_{sas}-Q_{sat}}{C_{sat}}, \\ \frac{dQ_{sat}}{dt} = \frac{P_{sat}-P_{svn}-(R_{sat}+R_{sar}+R_{scp})Q_{sat}}{L_{sat}}, \\ P_{sat} - P_{sat,un} = \frac{1}{C_{sat}}(V_{sat} - V_{sat,un}), \end{cases} \tag{S20}$$

$$\begin{cases} \frac{dP_{svn}}{dt} = \frac{Q_{sat}-Q_{svn}}{C_{svn}} - \frac{1}{C_{svn}}\frac{dV_{svn,un}}{dt}, \\ Q_{svn} = \frac{P_{svn}-P_{ra}}{R_{svn}}, \\ P_{svn} - P_{svn,un} = \frac{1}{C_{svn}}(V_{svn} - V_{svn,un}), \end{cases} \tag{S21}$$

Pulmonary Circuit

$$\begin{cases} \frac{dP_{pas}}{dt} = \frac{Q_{po}-Q_{pas}}{C_{pas}}, \\ \frac{dQ_{pas}}{dt} = \frac{P_{pas}-P_{pat}-R_{pas}Q_{pas}}{L_{pas}}, \\ P_{pas} - P_{pas,un} = \frac{1}{C_{pas}}(V_{pas} - V_{pas,un}) \end{cases} \tag{S22}$$

$$\begin{cases} \frac{dP_{pat}}{dt} = \frac{Q_{pas}-Q_{pat}}{C_{pat}}, \\ \frac{dQ_{pat}}{dt} = \frac{P_{pat}-P_{pvn}-(R_{pat}+R_{par}+R_{pcp})Q_{pat}}{L_{pat}}, \\ P_{pat} - P_{pat,un} = \frac{1}{C_{pat}}(V_{pat} - V_{pat,un}), \end{cases} \tag{S23}$$

$$\begin{cases} \frac{dP_{pvn}}{dt} = \frac{Q_{pat}-Q_{pvn}}{C_{pvn}}, \\ Q_{pvn} = \frac{P_{pvn}-P_{la}}{R_{pvn}}, \\ P_{pvn} - P_{pvn,un} = \frac{1}{C_{pvn}}(V_{pvn} - V_{pvn,un}). \end{cases} \tag{S24}$$

Baroreceptor Mechanism Equations

$$\begin{cases} \frac{dE_{lv,max}}{dt} = \frac{1}{\tau_h}\left(-E_{lv,max} + \alpha_{E_{lv}} n_s + \gamma_{E_{lv}}\right), \\ \frac{dE_{rv,max}}{dt} = \frac{1}{\tau_h}\left(-E_{rv,max} + \alpha_{E_{rv}} n_s + \gamma_{E_{rv}}\right), \end{cases} \tag{S25}$$

$$\begin{cases} \frac{dR_{sar}}{dt} = \frac{1}{\tau_a}\left(-R_{sar} + \alpha_{R_{sar}} n_s + \gamma_{R_{sar}}\right), \\ \frac{dR_{scp}}{dt} = \frac{1}{\tau_a}\left(-R_{scp} + \alpha_{R_{scp}} n_s + \gamma_{R_{scp}}\right), \end{cases} \tag{S26}$$

$$\begin{cases} \frac{dC_{svn}}{dt} = \frac{1}{\tau_v}\left(-C_{svn} + \alpha_{C_{svn}} n_s + \gamma_{C_{svn}}\right), \\ \frac{dV_{svn,un}}{dt} = \frac{1}{\tau_v}\left(-V_{svn,un} + \alpha_{V_{svn}} n_s + \gamma_{V_{svn}}\right). \end{cases} \tag{S27}$$

The sympathetic, n_s, and parasympathetic, n_p, activities [Hz] are expressed as

$$\begin{cases} n_s(\overline{P}_{cs}) = \frac{1}{1+\left(\frac{\overline{P}_{cs}}{\mu}\right)^{\nu}}, \\ n_p(\overline{P}_{cs}) = \frac{1}{1+\left(\frac{\overline{P}_{cs}}{\mu}\right)^{-\nu}}, \end{cases} \tag{S28}$$

where $\overline{P}_{cs}$ is the carotid sinus pressure averaged over one cardiac cycle, which is here taken equal to systemic arterial pressure averaged per beat, $\overline{P}_a$ [mmHg s]. μ [mmHg] is the mean systemic arterial pressure in physiological conditions at steady state for HR=70 bpm, while ν is a dimensionless parameter characterizing the steepness of the curves.

Initial Conditions and Cardiovascular Parameters

Initial conditions, which are given in terms of pressures, volumes, flow rates, valve opening angles and hemodynamic variables involved in the baroreceptor mechanisms, are the same for the three simulations: increased HRV, baseline, and decreased HRV. The total volume is taken as the typical value for a healthy adult, here V_{tot}=5246.42 ml. Flow rates and valve angles are initially considered as all the valves are closed and no flow is present. Initial pressures in the circulatory sections are chosen in the range reached during a normal cardiac cycle. Volumes in the four chambers are obtained at t=0 subtracting from the total volume, V_{tot}, all the volume contributes at the generic vascular section i, using the constitutive relation $P_{i,t=0} - P_{i,un} = \frac{1}{C_i}(V_{i,t=0} - V_{i,un})$, where $P_{i,t=0}$ is imposed as previously said. Table S1 summarizes the adopted initial values. Cardiovascular parameters of the model are as well recalled in Tables from S2 to S7.

CEREBRAL MODEL

The lumped model, proposed by Ursino and Giannessi [5], extends the Windkessel approach to the arterial and venous cerebral circulation, and is divided into three main parts: large arteries, distal arterial circulation, and capillary-venous circulation. The model is composed by a network of compliances, C, and resistances, R. The viscous effects are taken into account by the resistances, R [mmHg s/ml], while the elastic vessel properties are described by the compliances, C [ml/mmHg]. Three cardiovascular variables are involved at each section: the blood flow, Q [ml/s], the volume, V [ml], the pressure, P [mmHg].

Equations

For the sake of simplicity, the equations are grouped following the three main partitions of the model. The autoregulation and CO_2 activity equations for the distal district are separately reported.

Large arteries

$$\begin{cases} C_{ICA,left}\left(\dfrac{dP_{MCA,left}}{dt}-\dfrac{dP_{ic}}{dt}\right)=Q_{ICA,left}+Q_{PCoA,left}-Q_{MCA,left}-Q_{ACA1,left},\\ C_{ICA,right}\left(\dfrac{dP_{MCA,right}}{dt}-\dfrac{dP_{ic}}{dt}\right)=Q_{ICA,right}+Q_{PCoA,right}-Q_{MCA,right}-Q_{ACA1,right},\\ C_{BA}\dfrac{dP_{BA,willis}}{dt}=Q_{BA}-Q_{PCA1,left}-Q_{PCA1,right}, \end{cases} \tag{S29}$$

$$\begin{cases} Q_{ACA2,left}=Q_{ACA1,left}+Q_{ACoA},\\ Q_{ACA2,right}=Q_{ACA1,right}-Q_{ACoA},\\ Q_{PCA2,left}=-Q_{PCoA,left}+Q_{PCA1,left},\\ Q_{PCA2,right}=-Q_{PCoA,right}+Q_{PCA1,right}, \end{cases} \tag{S30}$$

where the flow rates, Q, are:

$$\begin{cases} Q_{ICA,left}=\dfrac{P_a-P_{MCA,left}}{R_{ICA,left}},\\ Q_{ICA,right}=\dfrac{P_a-P_{MCA,right}}{R_{ICA,right}},\\ Q_{BA}=\dfrac{P_a-P_{BA,willis}}{R_{BA}}, \end{cases} \tag{S31}$$

$$\begin{cases} Q_{MCA,left} = \dfrac{P_{MCA,left} - P_{dm,left}}{R_{MCA,left} + R_{dm,left}/2}, \\ Q_{MCA,right} = \dfrac{P_{MCA,right} - P_{dm,right}}{R_{MCA,right} + R_{dm,right}/2}, \\ Q_{ACA1,left} = \dfrac{P_{ICA,left} - P_{ACA,left}}{R_{ACA1,left}}, \\ Q_{ACA1,right} = \dfrac{P_{ICA,right} - P_{ACA,right}}{R_{ACA1,right}}, \\ Q_{PCA1,left} = \dfrac{P_{BA,willis} - P_{PCA,left}}{R_{PCA1,left}}, \\ Q_{PCA1,right} = \dfrac{P_{BA,willis} - P_{PCA,right}}{R_{PCA1,right}}, \end{cases} \tag{S32}$$

$$\begin{cases} Q_{ACA2,left} = \dfrac{P_{ACA,left} - P_{da,left}}{R_{ACA2,left} + R_{da,left}/2}, \\ Q_{ACA2,right} = \dfrac{P_{ACA,right} - P_{da,right}}{R_{ACA2,right} + R_{da,right}/2}, \\ Q_{ACoA} = \dfrac{P_{ACA,right} - P_{ACA,left}}{R_{ACoA}}, \\ Q_{PCA2,left} = \dfrac{P_{PCA,left} - P_{dp,left}}{R_{PCA2,left} + R_{dp,left}/2}, \\ Q_{PCA2,right} = \dfrac{P_{PCA,right} - P_{dp,right}}{R_{PCA2,right} + R_{dp,right}/2}, \\ Q_{PCoA,left} = \dfrac{P_{PCA,left} - P_{MCA,left}}{R_{PCoA,left}}, \\ Q_{PCoA,right} = \dfrac{P_{PCA,right} - P_{MCA,right}}{R_{PCoA,right}}, \end{cases} \tag{S33}$$

Distal arterial circulation

$$\begin{cases} \dfrac{dV_{dm,left}}{dt} = Q_{MCA,left} - Q_{dm,left} + Q_{cam,left} + Q_{cpm,left}, \\ \dfrac{dV_{dm,right}}{dt} = Q_{MCA,right} - Q_{dm,right} + Q_{cam,right} + Q_{cpm,right}, \\ \dfrac{dV_{da,left}}{dt} = Q_{ACA2,left} - Q_{da,left} - Q_{cam,left} + Q_{caa}, \\ \dfrac{dV_{da,right}}{dt} = Q_{ACA2,right} - Q_{da,right} - Q_{cam,right} - Q_{caa}, \\ \dfrac{dV_{dp,left}}{dt} = Q_{PCA2,left} - Q_{dp,left} - Q_{cpm,left} + Q_{cpp}, \\ \dfrac{dV_{dp,right}}{dt} = Q_{PCA2,right} - Q_{dp,right} - Q_{cpm,right} - Q_{cpp}, \end{cases} \tag{S34}$$

where the flow rates, Q, are:

$$\begin{cases} Q_{dm,left} = \dfrac{P_{dm,left} - P_c}{R_{dm,left}/2}, \\ Q_{dm,right} = \dfrac{P_{dm,right} - P_c}{R_{dm,right}/2}, \\ Q_{da,left} = \dfrac{P_{da,left} - P_c}{R_{da,left}/2}, \\ Q_{da,right} = \dfrac{P_{da,right} - P_c}{R_{da,right}/2}, \\ Q_{dp,left} = \dfrac{P_{dp,left} - P_c}{R_{dp,left}/2}, \\ Q_{dp,right} = \dfrac{P_{dp,right} - P_c}{R_{dp,right}/2}, \end{cases} \tag{S35}$$

$$\begin{cases} Q_{cam,left} = \dfrac{P_{da,left} - P_{dm,left}}{R_{cam,left}}, \\ Q_{cam,right} = \dfrac{P_{da,right} - P_{dm,right}}{R_{cam,right}}, \\ Q_{cpm,left} = \dfrac{P_{dp,left} - P_{dm,left}}{R_{cpm,left}}, \\ Q_{cpm,right} = \dfrac{P_{dp,right} - P_{dm,right}}{R_{cpm,right}}, \\ Q_{caa} = \dfrac{P_{da,right} - P_{da,left}}{R_{caa}}, \\ Q_{cpp} = \dfrac{P_{dp,right} - P_{dp,left}}{R_{cpp}}, \end{cases} \tag{S36}$$

while the constitutive relations are:

$$\begin{cases} P_{dm,left} = \dfrac{V_{dm,left}}{C_{dm,left}} + P_{ic}, \\ P_{dm,right} = \dfrac{V_{dm,right}}{C_{dm,right}} + P_{ic}, \\ P_{da,left} = \dfrac{V_{da,left}}{C_{da,left}} + P_{ic}, \\ P_{da,right} = \dfrac{V_{da,right}}{C_{da,right}} + P_{ic}, \\ P_{dp,left} = \dfrac{V_{dp,left}}{C_{dp,left}} + P_{ic}, \\ P_{dp,right} = \dfrac{V_{dp,right}}{C_{dp,right}} + P_{ic}, \end{cases} \tag{S37}$$

Capillary-venous circulation

$$\begin{cases} C_{ic}\dfrac{dP_{ic}}{dt} = Q_{MCA,left} - Q_{dm,left} + Q_{PCA2,left} - Q_{dp,left} + Q_{ACA2,left} - Q_{da,left} \\ \quad + Q_{MCA,right} - Q_{dm,right} + Q_{PCA2,right} - Q_{dp,right} + Q_{ACA2,right} - Q_{da,right} \\ \quad + Q_{pv} - Q_{vs} + Q_f - Q_o, \\ C_{vi}\left(\dfrac{dP_v}{dt} - \dfrac{dP_{ic}}{dt}\right) = Q_{pv} - Q_{vs}, \\ Q_{dm,left} + Q_{da,left} + Q_{dp,left} + Q_{dm,right} + Q_{da,right} + Q_{dp,right} = Q_f + Q_{pv}, \end{cases} \tag{S38}$$

where the flow rates, Q, the compliances C_{ic}, C_{vi}, and the resistance R_{vs} are defined as follows:

$$\begin{cases} Q_{pv} = \dfrac{P_c - P_v}{R_{pv}}, \\ Q_{vs} = \dfrac{P_v - P_{vs}}{R_{vs}}, \\ Q_f = \begin{cases} \dfrac{P_c - P_{ic}}{R_f}, & \text{if} \quad P_c \geq P_{ic}, \\ 0, & \text{if} \quad P_c < P_{ic}, \end{cases} \\ Q_o = \begin{cases} \dfrac{P_{ic} - P_{vs}}{R_o}, & \text{if} \quad P_{ic} \geq P_{vs}, \\ 0, & \text{if} \quad P_{ic} < P_{vs}, \end{cases} \\ Q_{out} = Q_{vs} + Q_o, \end{cases} \tag{S39}$$

$$\begin{cases} C_{ic} = \dfrac{1}{k_E P_{ic}}, \\ C_{vi} = \dfrac{1}{k_{ven}(P_v - P_{ic} - P_{v1})}, \\ R_{vs} = \begin{cases} \dfrac{P_v - P_{vs}}{P_v - P_{ic}} R_{vs1}, & \text{if} \quad P_{vs} < P_{ic}, \\ R_{vs1}, \quad \text{if} \quad P_{vs} \geq P_{ic}, \end{cases} \end{cases} \tag{S40}$$

Autoregulation and CO_2 reactivity equations

For each of the six distal regions, the following equations hold:

$$\begin{cases} \tau_{aut} \dfrac{dx_{aut,i,j}}{dt} = -x_{aut,i,j} + G_{aut}\left(\dfrac{Q_{di,j} - Q_{ndi,j}}{Q_{ndi,j}}\right), \quad \text{i=m,a,p; j=left,right}, \\ \tau_{CO_2} \dfrac{dx_{CO_2,i,j}}{dt} = -x_{CO_2,i,j} + G_{CO_2} A_{CO_2,i,j} \log_{10}\left(\dfrac{P_{aCO_2}}{P_{aCO_2n}}\right), \quad \text{i=m,a,p; j=left,right}, \end{cases} \tag{S41}$$

where the subscript n denotes the basal values and

$$A_{CO_2,i,j} = \frac{1}{1 + \exp\{[-k_{CO_2}(Q_{di,j} - Q_{ndi,j})/Q_{ndi,j}] - b_{CO_2}\}}, \quad \text{i=m,a,p; j=left,right.} \tag{S42}$$

Distal compliances and resistances are ruled by the following relations:

$$\begin{cases} C_{di,j} = \dfrac{C_{d0i,j}\left[(1 - \Delta C_{di,j}/2) + (1 + \Delta C_{di,j}/2)\exp\left(\frac{x_{CO_2,i,j} - x_{aut,i,j}}{k_{C_{di,j}}}\right)\right]}{1 + \exp[(x_{CO_2,i,j} - x_{aut,i,j})/k_{C_{di,j}}]}, \\ R_{di,j} = \dfrac{k_{R_{di,j}} C_{d0i,j}^2}{V_{di,j}^2}, \quad \text{i=m,a,p; j=left,right}, \end{cases} \tag{S43}$$

with

$$\begin{aligned} \Delta C_{di,j} &= \begin{cases} 2s_1, & \text{if} \quad x_{CO_2,i,j} < x_{aut,i,j}, \\ 2s_2, & \text{if} \quad x_{CO_2,i,j} \geq x_{aut,i,j}, \end{cases} \quad \text{i=m,a,p; j=left,right} \\ k_{C_{di,j}} &= \begin{cases} \dfrac{C_{d0i,j} s_1}{2}, & \text{if} \quad x_{CO_2,i,j} < x_{aut,i,j}, \\ \dfrac{C_{d0i,j} s_2}{2}, & \text{if} \quad x_{CO_2,i,j} \geq x_{aut,i,j}, \end{cases} \quad \text{i=m,a,p; j=left,right} \end{aligned} \tag{S44}$$

Initial Conditions and Cerebral Parameters

Initial conditions are given in terms of pressures, volumes, autoregulation and CO_2 reactivity state variables, and are equal in the three HRV configurations: increased HRV, baseline, and decreased HRV. The initial setting is chosen according to flow rate repartition given by [5] with the following total basal values: basal cerebral blood flow $Q_n = 12.5$ ml/s, basal distal resistance $R_{dn} = 5.4$ mmHg s/ml, basal distal compliance $C_{dn} = 0.2$ ml/mmHg. State variables for autoregulation, x_{aut}, and CO_2 reactivity, x_{CO_2}, are initially considered as no activity is present. Initial pressures in the cerebral regions are chosen in the physiological range reached during a normal cardiac cycle. Volumes in the distal region j are obtained at t=0 using the constitutive relation $V_{dj,t=0} = C_{dj,t=0}(P_{dj,t=0} - P_{ic,t=0})$, where $P_{dj,t=0}$ and $P_{ic,t=0}$ are imposed as previously said. The adopted initial conditions are listed in Table S8, while parameters of the cerebral model are reported in Tables from S9 to S12.

HEART FAILURE

A heart failure condition of level III according to the criteria of American New York Heart Association [6] is considered, by decreasing the ventricular contractilities ($E_{lv,max}$ -80%, $E_{rv,max}$ -40%), increasing systemic (R_{sar} and R_{scp} +25%) and pulmonary (R_{par} and R_{pcp} +40%) arterial resistances, and increasing the total blood volume (+ 300 ml) [7–10]. The adopted values for these parameters are reported in Table S13. We assumed that the cerebral flow autoregulation function is preserved as in the healthy case. The resulting simulated hemodynamic variables for the baseline heart failure condition are listed in Table S14, and show a very good matching with clinical literature data [11–20]. The only exception is related to pulmonary arterial pressure parameters, which increase in agreement with measured data but are underestimated.

To define the increased and decreased HRV configurations, we adopted the exponential relation:

$$\mathrm{SDNN} = 309.4\mathrm{e}^{-0.021\mathrm{HR}} \tag{S45}$$

similarly to the healthy case. The baseline configuration was set at RR= 0.70 [s], HR= 85.29 bpm according to the steady state baseline results (see Table S14), and SDNN=51.60 [ms] was obtained from the above relation (S45). Increased and decreased HRV configurations were achieved by changing SDNN by +20% and -20% (with consequent HR variation through Eq. (S45)), obtaining the configurations reported in Table S15.

NUMERICAL SCHEME

The differential equations of both models were solved by means of numerical algorithms implemented in Matlab, which use a multistep adaptive solver through the `ode15s` function. This variable order solver is based on the numerical differentiation formulas (NDFs) and is chosen because is one of the most efficient and suitable routines for stiff problems. In fact, the differential system of both models shows some stiffness features, that is the equations include some terms that can lead to rapid variation in the solutions. This aspect is particularly relevant during end-diastolic and end-systolic phases, when the valves opening and closure cause sudden variations of the leaflet angular position. The Matlab scripts as well as the datasets generated and analyzed during the current study are available from the corresponding author upon request.

REFERENCES

[1] Scarsoglio S, Guala A, Camporeale C, Ridolfi L. Impact of atrial fibrillation on the cardiovascular system through a lumped-parameter approach. *Med. Biol. Eng. Comput.* **52** (2014) 905–920. doi:10.1007/s11517-014-1192-4.

[2] Scarsoglio S, Camporeale C, Guala A, Ridolfi L. Fluid dynamics of heart valves during atrial fibrillation: a lumped parameter-based approach. *Comput. Methods Biomech. Biomed. Engin.* **19** (2016) 1060–1068. doi:10.1080/10255842.2015.1094800.

[3] Korakianitis T, Shi Y. Numerical simulation of cardiovascular dynamics with healthy and diseased heart valves. *J. Biomech.* **39** (2006) 1964–1982. doi:10.1016/j.jbiomech.2005.06.016.

[4] Ottesen J, Olufsen M, Larsen J. *Applied Mathematical Models in Human Physiology* (Philadelphia, Pensylvania: SIAM) (2004).

[5] Ursino M, Giannessi M. A model of cerebrovascular reactivity including the circle of Willis and cortical anastomoses. *Ann. Biomed. Eng.* **38** (2010) 955–74. doi:10.1007/s10439-010-9923-7.

[6] of the New York Heart Association TCC. *Nomenclature and Criteria for Diagnosis of Diseases of the Heart and Great Vessels* (Boston: Little, Brown & Co) (1994).

[7] Lim E, Chan G, Dokos S, Ng S, Latif L, Vandenberghe S, et al. A cardiovascular mathematical model of graded head-up tilt. *PLoS One* **8** (2013) e77357. doi:10.1371/journal.pone.0077357.

[8] Bozkurt S, Safak K. Evaluating the hemodynamical response of a cardiovascular system under support of a continuous flow left ventricular assist device via numerical modeling and simulations. *Comput. Math. Methods Med.* **2013** (2013) 12. doi:10.1155/2013/986430.

[9] Bozkurt S. Mathematical modeling of cardiac function to evaluate clinical cases in adults and children. *PLoS One* **14** (2019) e0224663. doi:10.1371/journal.pone.0224663.

[10] Zhu S, Luo L, Yang B, Li X, Wang X. Improving hemodynamics of cardiovascular system under a novel intraventricular assist device support via modeling and simulations. *Comput. Assist. Surg.* **22** (2017) 221–231. doi:10.1080/24699322.2017.1389400.

[11] Beltrami C, Finato N, Rocco M, Feruglio G, Puricelli C, Cigola E, et al. Structural basis of end-stage failure in ischemic cardiomyopathy in humans. *Circulation* **89** (1994) 151–63. doi:10.1161/01.cir.89.1.151.

[12] Burkhoff D, Flaherty J, Yue D, Herskowitz A, Oikawa R, Sugiura S, et al. In vitro studies of isolated supported human hearts. *Heart Vessels* **4** (1988) 185–196. doi:10.1007/BF02058586.

[13] Felix S, Staudt A, Dorffel W, Stangl V, Merkel K, Pohl M, et al. Hemodynamic effects of immunoadsorption and subsequent immunoglobulin substitution in dilated cardiomyopathy: three-month results from a randomized study. *J. Am. Coll. Cardiol.* **35** (2000) 1590–8. doi:10.1016/s0735-1097(00)00568-4.

[14] Hobbs R, Miller L, Bott-Silverman C, James K, Rincon G, Grossbard E. Hemodynamic effects of a single intravenous injection of synthetic human brain natriuretic peptide in patients with heart failure secondary to ischemic or idiopathic dilated cardiomyopathy. *Am. J. Cardiol.* **78** (1996) 896–901. doi:10.1016/s0002-9149(96)00464-x.

[15] Leclercq C, Cazeau S, Breton HL, Ritter P, Mabo P, Gras D, et al. Acute hemodynamic effects of biventricular ddd pacing in patients with end-stage heart failure. *J. Am. Coll. Cardiol.* **32** (1998) 1825–31. doi:10.1016/s0735-1097(98)00492-6.

[16] Makarenko I, Opitz C, Leake M, Neagoe C, Kulke M, Gwathmey J, et al. Passive stiffness changes caused by upregulation of compliant titin isoforms in human dilated cardiomyopathy hearts. *Circ Res.* **95** (2004) 708–16. doi:10.1161/01.RES.0000143901.37063.2f.

[17] Pak P, Maughan L, Baughman K, Kass D. Marked discordance between dynamic and passive diastolic pressure-volume relations in idiopathic hypertrophic cardiomyopathy. *Circulation* **94** (1996) 52–60. doi:10.1161/01.cir.94.1.52.

[18] Sato H, Hori M, Ozaki H, Yokoyama H, Imai K, Morikawa M, et al. Exercise-induced upward shift of diastolic left ventricular pressure-volume relation in patients with dilated cardiomyopathy. effects of beta-adrenoceptor blockade. *Circulation* **88** (1993) 2215–23. doi:10.1161/01.cir.88.5.2215.

[19] Stolf N, Moreira L, Bocchi E, Higuchi M, Bacal F, Bellotti G, et al. Determinants of midterm outcome of partial left ventriculectomy in dilated cardiomyopathy. *Ann. Thorac. Surg.* **66** (1998) 1585–91. doi:10.1016/s0003-4975(98)00959-x.

[20] Waagstein F, Caidahl K, Wallentin I, Bergh C, Hjalmarson A. Long-term beta-blockade in dilated cardiomyopathy. effects of short- and long-term metoprolol treatment followed by withdrawal and readministration of metoprolol. *Circulation* **80** (1989) 551–63. doi:10.1161/01.cir.80.3.551.

[21] Korakianitis T, Shi Y. A concentrated parameter model for the human cardiovascular system including heart valve dynamics and atrioventricular interaction. *Med. Eng. Phys.* **28** (2006) 613–628. doi:10.1016/j.medengphy.2005.10.004.

[22] Ruiz P, Rezaienia MA, Rahideh A, Keeble T, Rothman M, Korakianitis T. In vitro cardiovascular system emulator (bioreactor) for the simulation of normal and diseased conditions with and without mechanical circulatory support. *Artif. Organs* **37** (2013) 549–560. doi:10.1111/aor.12109.

[23] Guyton A, Hall J. *Textbook of Medical Physiology* (Philadelphia, Pensylvania: Elsevier Saunders) (2006).

Variable	Value ($t = 0$)
$V_{la,0}$	60 ml
$V_{lv,0}$	130.06 ml
$V_{ra,0}$	39 ml
$V_{rv,0}$	110 ml
$P_{sas,0}$	124.75 mmHg
$Q_{sas,0}$	0 ml/s
$P_{sat,0}$	124.75 mmHg
$Q_{sat,0}$	0 ml/s
$P_{svn,0}$	10 mmHg
$P_{pas,0}$	21.11 mmHg
$Q_{pas,0}$	0 ml/s
$P_{pat,0}$	21.11 mmHg
$Q_{pat,0}$	0 ml/s
$P_{pvn,0}$	10 mmHg
$\theta_{mi,0} = d\theta_{mi,0}/dt$	0 rad
$\theta_{ao,0} = d\theta_{ao,0}/dt$	0 rad
$\theta_{ti,0} = d\theta_{ti,0}/dt$	0 rad
$\theta_{po,0} = d\theta_{po,0}/dt$	0 rad
$E_{lv,max,0}$	2.5 mmHg/ml
$E_{rv,max,0}$	1.15 mmHg/ml
$R_{sar,0}$	0.44 mmHg s/ml
$R_{scp,0}$	0.4576 mmHg s/ml
$C_{svn,0}$	20.5 ml/mmHg
$V_{svn,un,0}$	3000 ml

Table S1. Cardiovascular model: initial conditions.

Parameter	Value
CQ_{ao}	350 ml/(s mmHg$^{0.5}$)
CQ_{mi}	400 ml/(s mmHg$^{0.5}$)
$E_{lv,min}$	0.07 mmHg/ml
$P_{lv,un}$	1 mmHg
$V_{lv,un}$	5 ml
$E_{la,max}$	0.25 mmHg/ml
$E_{la,min}$	0.15 mmHg/ml
$P_{la,un}$	1 mmHg
$V_{la,un}$	4 ml
CQ_{po}	350 ml/(s mmHg$^{0.5}$)
CQ_{ti}	400 ml/(s mmHg$^{0.5}$)
$E_{rv,min}$	0.07 mmHg/ml
$P_{rv,un}$	1 mmHg
$V_{rv,un}$	10 ml
$E_{ra,max}$	0.25 mmHg/ml
$E_{ra,min}$	0.15 mmHg/ml
$P_{ra,un}$	1 mmHg
$V_{ra,un}$	4 ml

Table S2. Cardiovascular model: heart parameters.

Parameter	Value
C_{sas}	0.064 ml/mmHg
R_{sas}	0.003 mmHg s/ml
L_{sas}	0.000062 mmHg s^2/ml
$P_{sas,un}$	1 mmHg
$V_{sas,un}$	25 ml
C_{sat}	1.28 ml/mmHg
R_{sat}	0.05 mmHg s/ml
L_{sat}	0.0017 mmHg s^2/ml
$P_{sat,un}$	1 mmHg
$V_{sat,un}$	775 ml
R_{svn}	0.075 mmHg s/ml
$P_{svn,un}$	1 mmHg

Table S3. Cardiovascular model: systemic circulation parameters.

Parameter	Value
C_{pas}	0.162 ml/mmHg
R_{pas}	0.002 mmHg s/ml
L_{pas}	0.000052 mmHg s^2/ml
$P_{pas,un}$	1 mmHg
$V_{pas,un}$	25 ml
C_{pat}	3.42 ml/mmHg
R_{pat}	0.01 mmHg s/ml
L_{pat}	0.0017 mmHg s^2/ml
$P_{pat,un}$	1 mmHg
$V_{pat,un}$	175 ml
R_{par}	0.0275 mmHg s/ml
R_{pcp}	0.0385 mmHg s/ml
R_{pvn}	0.006 mmHg s/ml
C_{pvn}	20.5 ml/mmHg
$P_{pvn,un}$	1 mmHg
$V_{pvn,un}$	300 ml

Table S4. Cardiovascular model: pulmonary circulation parameters.

Parameter	Value
$K_{p,mi}$, $K_{p,ao}$, $K_{p,ti}$, $K_{p,po}$	5500 rad/(s^2 mmHg)
$K_{f,mi}$, $K_{f,ao}$, $K_{f,ti}$, $K_{f,po}$	50 s^{-1}
$K_{b,mi}$, $K_{b,ao}$, $K_{b,ti}$, $K_{b,po}$	2 rad/(s ml)
$K_{v,mi}$, $K_{v,ti}$	3.5 rad/(s ml)
$K_{v,ao}$, $K_{v,po}$	7 rad/(s ml)
θ_{max}	5/12 π rad

Table S5. Cardiovascular model: valve dynamics parameters.

Parameter	Value
T_{ac}	0.875 RR s
T_{me}	0.3 $\sqrt{RR}$ s
T_{ce}	3/2 T_{me} s

Table S6. Cardiovascular model: temporal parameters. Activation times are those typically introduced in the time-varying elastance models ref [3, 4, 21, 22], and are here settled considering that, at RR=0.8 s, the ventricular systole lasts about 0.3 s, while the atrial systole length is about 0.1 s [23].

Parameter	Value
τ_h	3 s
τ_a	12 s
τ_v	60 s
μ	97.13 mmHg
ν	7
$\alpha_{E_{lv}}$	1 mmHg s/ml
$\gamma_{E_{lv}}$	2 mmHg/ml
$\alpha_{E_{rv}}$	0.46 mmHg s/ml
$\gamma_{E_{rv}}$	0.92 mmHg/ml
$\alpha_{R_{sar}}$	0.3520 mmHg s^2/ml
$\gamma_{R_{sar}}$	0.2640 mmHg s/ml
$\alpha_{R_{scp}}$	0.3660 mmHg s^2/ml
$\gamma_{R_{scp}}$	0.2746 mmHg s/ml
$\alpha_{C_{svn}}$	-4.1 ml s/mmHg
$\gamma_{C_{svn}}$	22.55 ml/mmHg
$\alpha_{V_{svn}}$	-1260 ml s
$\gamma_{V_{svn}}$	3630 ml

Table S7. Cardiovascular model: baroreceptor parameters. Time delays (τ_h: heart, τ_a: arterial, τ_v: vein) and coefficients (α_i, γ_i, μ, ν) for the strength of the sympathetic and parasympathetic activities are taken according to the validated model by [4].

Variable	Value ($t = 0$)
$P_{ic,0}$	9.5 mmHg
$P_{v,0}$	14 mmHg
$P_{dm,left,0}$	58.5 mmHg
$V_{dm,left,0}$	2.94 ml
$P_{dm,right,0}$	58.5 mmHg
$V_{dm,right,0}$	2.94 ml
$P_{da,left,0}$	58.5 mmHg
$V_{da,left,0}$	0.78 ml
$P_{da,right,0}$	58.5 mmHg
$V_{da,right,0}$	0.78 ml
$P_{dp,left,0}$	58.5 mmHg
$V_{dp,left,0}$	1.18 ml
$P_{dp,right,0}$	58.5 mmHg
$V_{dp,right,0}$	1.18 ml
$P_{ICA,left,0}$	100 mmHg
$P_{ICA,right,0}$	100 mmHg
$P_{BA,willis,0}$	100 mmHg
$x_{aut,i,j,0}$<br>i=m,a,p, j=left,right	0
$x_{CO_2,i,j,0}$<br>i=m,a,p, j=left,right	0

Table S8. Cerebral model: initial conditions.

Parameter	Value
$R_{ICA,left}$	0.5689 mmHg s/ml
$R_{ICA,right}$	0.5689 mmHg s/ml
R_{BA}	0.4501 mmHg s/ml
$R_{MCA,left}$	1.4419 mmHg s/ml
$R_{MCA,right}$	1.4419 mmHg s/ml
$R_{PCA1,left}$	0.7640 mmHg s/ml
$R_{PCA1,right}$	0.7640 mmHg s/ml
$R_{ACA1,left}$	3.7912 mmHg s/ml
$R_{ACA1,right}$	3.7912 mmHg s/ml
$R_{PCA2,left}$	3.6063 mmHg s/ml
$R_{PCA2,right}$	3.6063 mmHg s/ml
$R_{ACA2,left}$	1.6227 mmHg s/ml
$R_{ACA2,right}$	1.6227 mmHg s/ml
$R_{PCoA,left}$	90.9786 mmHg s/ml
$R_{PCoA,right}$	90.9786 mmHg s/ml
R_{ACoA}	14.9228 mmHg s/ml
$C_{ICA,left}$	0.0034 ml/mmHg
$C_{ICA,right}$	0.0034 ml/mmHg
C_{BA}	0.0017 ml/mmHg

Table S9. Cerebral model: large arteries parameters.

Parameter	Value
$R_{cam,left}$	105 mmHg s/ml
$R_{cam,right}$	105 mmHg s/ml
$R_{cpm,left}$	120 mmHg s/ml
$R_{cpm,right}$	120 mmHg s/ml
R_{caa}	22 mmHg s/ml
R_{cpp}	75 mmHg s/ml

Table S10. Cerebral model: distal arterial circulation parameters.

Parameter	Value
R_f	$2.3 \cdot 10^3$ mmHg s/ml
R_o	526.3 mmHg s/ml
R_{pv}	0.880 mmHg s/ml
R_{vs1}	0.366 mmHg s/ml
k_E	0.077 ml^{-1}
k_{ven}	0.155 ml^{-1}
P_{vs}	6 mmHg
P_{v1}	-2.5 mmHg

Table S11. Cerebral model: capillary-venous circulation parameters.

Parameter	Value
τ_{aut}	20 s
G_{aut}	0.9
τ_{CO_2}	40 s
G_{CO_2}	4.0
k_{CO_2}	15
b_{CO_2}	0.5
P_{aCO_2n}	40 mmHg
P_{aCO_2}	40 mmHg
s_1	7
s_2	0.4
$Q_{ndm,left}$	3.75 ml/s
$Q_{ndm,right}$	3.75 ml/s
$Q_{nda,left}$	1 ml/s
$Q_{nda,right}$	1 ml/s
$Q_{ndp,left}$	1.5 ml/s
$Q_{ndp,right}$	1.5 ml/s
$k_{R_{dm,left}}$	$4.2848 \cdot 10^{4}$ mmHg^{-3} s/ml
$k_{R_{dm,right}}$	$4.2848 \cdot 10^{4}$ mmHg^{-3} s/ml
$k_{R_{da,left}}$	$1.6060 \cdot 10^{5}$ mmHg^{-3} s/ml
$k_{R_{da,right}}$	$1.6060 \cdot 10^{5}$ mmHg^{-3} s/ml
$k_{R_{dp,left}}$	$1.0777 \cdot 10^{5}$ mmHg^{-3} s/ml
$k_{R_{dp,right}}$	$1.0777 \cdot 10^{5}$ mmHg^{-3} s/ml
$C_{d0m,left}$	0.06 ml/mmHg
$C_{d0m,right}$	0.06 ml/mmHg
$C_{d0a,left}$	0.016 ml/mmHg
$C_{d0a,right}$	0.016 ml/mmHg
$C_{d0p,left}$	0.024 ml/mmHg
$C_{d0p,right}$	0.024 ml/mmHg

Table S12. Cerebral model: autoregulation and CO_2 reactivity parameters.

Parameter	Value
$\alpha_{E_{lv}}$	0.2 mmHg s/ml
$\gamma_{E_{lv}}$	0.4 mmHg/ml
$\alpha_{E_{rv}}$	0.2760 mmHg s/ml
$\gamma_{E_{rv}}$	0.5520 mmHg/ml
$\alpha_{R_{sar}}$	0.44 mmHg s^2/ml
$\gamma_{R_{sar}}$	0.33 mmHg s/ml
$\alpha_{R_{scp}}$	0.4576 mmHg s^2/ml
$\gamma_{R_{scp}}$	0.3432 mmHg s/ml
R_{par}	0.0385 mmHg s/ml
R_{pcp}	0.0539 mmHg s/ml
V_{tot}	5546.42 ml

Table S13. Heart failure. Cardiovascular model parameters modified for the baseline configuration.

Table S14. Heart failure. Modeled hemodynamic parameters for the baseline configuration and comparison with clinical literature [11–20].

**Variable**	**Baseline model**	**Literature measured data**
$P_{a,syst}$ [mmHg]	106.34	107–115
$P_{a,dias}$ [mmHg]	75.50	68–76
$\bar{P}_a$ [mmHg]	89.14	78–95
$P_{pas,syst}$ [mmHg]	29.76	54–62
$P_{pas,dias}$ [mmHg]	20.19	28–29
$\bar{P}_{pas}$ [mmHg]	24.24	27–40
$\bar{P}_{la}$ [mmHg]	17.71	17–29
HR [bpm]	85.29	76–103
V_{lved} [ml]	253.13	205–522
V_{lves} [ml]	211.55	140–249
SV [ml]	41.58	32.40-50.40
EF [%]	16.43	15–33
CO [l/min]	3.55	3.42-4.32

**Configuration**	$SDNN$ **[ms]**	RR **[s]**	HR **[bpm]**
Increased HRV (+20%)	61.92	0.78	76.61
Baseline	51.60	0.70	85.29
Decreased HRV (-20%)	41.28	0.63	95.91

Table S15. Heart failure. SDNN, RR and HR values of the three HRV configurations.
